# Review

**Published:** 1897-08

**Authors:** 


### REVIEW.

The Report of the Committee Appointed by the Massachusetts Legislature to Examine into the Matter of Tubebculosis of Cattle. House Document 1341. Boston, Mass.

On April 9th of the present year a joint special committee of the Massachusetts Legislature was appointed to investigate and report upon the post-mortem examinations of certain cows from Lowell and Dracut that were condemned as tuberculous after the application of the tuberculin-test. This committee was made up of eleven members from the House and Senate, and their report was published on May 25, 1897. They employed as experts to assist in the examination of these animals Dr. H. C. Ernst, of Boston; Dr. Theobald Smith, of Boston; Dr. George N. Kinnell, of Pittsfield; Dr. Charles R. Wood, of Lowell, and Dr. Frank S. Billings, of Grafton. It seems that 150 cattle in the vicinity of Lowell and Dracut were examined by a private

**Rest is often found in a change of scenes and among your old acquaintances.**

firm of veterinarians. Of these 128 responded to the test in such a manner that they were condemned as tuberculous, and 2 (?) were condemned as tuberculous on physical examination. The discovery of such a large number of tuberculous animals in herds that were not known to be seriously infected caused considerable alarm, and one of the members of the Legislature from the district in which the diseased cattle were found proposed that the committee, as above referred to, should be appointed to make a thorough investigation of the matter, and this was done with the assistance of the experts named above.

The publication contains a majority report signed by nine members of the committee, and a minority report signed by two members of the committee, together with a separate report from each of the experts. The reports of the experts furnish the complete facts in reference to the post-mortem conditions found, and also include summaries of the work done and various pertinent and impertinent suggestions. The experts agree in the main as to the post-mortem lesions, and the facts in reference to the work as compiled from the various reports seem to be as follows:

**Not less than ten colleges will be represented at Nashville. Your old teachers may be among them.**

Of the 130 animals condemned, 128 responded to the tuberculin-test, and 2 (?) were condemned on physical examination. The post-mortem examination of the 130 cattle showed that 126 of those condemned by the tuberculin-test were actually tuberculous, while the other 2 did not show any evidence of tuberculosis that could be detected by the naked eye, and glands were reserved for microscopic examination, which will be reported upon later. The two condemned on physical examination were found to be free from tuberculosis when slaughtered. In order to ascertain with what degree of thoroughness the disease had been eradicated from the remaining 20 animals that were not condemned, these were also purchased by the State and killed. It is to be observed that the 20 animals belonging to this category were kept in the infected premises occupied by the diseased herds for from between five and six weeks after they were examined, and although a second test prior to the slaughter was suggested by some of the experts and members of the committee, it was not made.

Upon post-mortem examination 6 of these 20 animals were found to contain the lesions of tuberculosis, the extent of which is shown by the following quotations from the report of Dr. Harold C. Ernst: "Cow No. 2, a number of fairly large, broken-down tuberculous nodules in the right tracheal gland; multiple tubercles in one mediastinal gland. Cow No. 3, multiple tuberculous foci (small) in left bronchial gland and in upper mediastinal gland; a single small tubercle in right caudal lobe, a flat suspicious-looking nodule on external muscular surface of the abdomen.

**On to Nashville and the "Sunny South."**

Cow No. 14, minute tubercles in both mediastinal glands, a small one in the right caudal lobe. Cow No. 15, a single calcified pinhead nodule in one left bronchial gland. Cow No. 18, several minute tubercles in posterior mediastinal glands." The reports of Dr. Theobald Smith and the other experts agree with the above. In reference to this series of post-mortems Dr. Ernst remarks that "it is unfortunate that they were not all re-tested just before the slaughtering, because it is quite open to question whether the infection did not occur after the first injection of tuberculin. *In none of them was there any extensive infection.*" In four the lesions were confined exclusively to lymphatic glands, while lesions existed in the lymphatic glands in the other two, and there were also single tubercular foci in the lungs, one in each animal. Of the 128 animals condemned with tuberculin the report shows a much higher degree of infection in most of them than was observed in the 6 tuberculous animals that were not condemned. Some of them, in fact, were very extensively diseased, and the lesions were found widely distributed throughout the body. Infection of the udder was not noticed in any case, although in two cases the lymph glands of the udder were involved.

Based upon these investigations the majority of the committee makes various recommendations, of which the three following are of the greatest importance and interest:

1. Attention is called to the necessity of thoroughly cleansing and disinfecting the buildings in which condemned animals have been kept, and this, it is stated, should be done at the earliest opportunity and under the order or supervision of the Board of Cattle Commissioners, or their agents, and before other animals are placed therein.

If this recommendation is based upon the experience of the committee there is every reason to believe that it is in effect a statement that they know of cows that have probably become infected from infected surroundings, and a tacit admission that such may have been the case in some of the six tuberculous cattle that were not condemned by the test made between five and six weeks before the animals were killed.

2. It is stated that a large proportion of the tuberculous animals that were killed were so slightly affected as to warrant the use of their meat for food if slaughtered and dressed under proper supervision. This may justly be regarded as one of the most important results of the investigation, and it is to be hoped that this matter will remain under consideration, and that in time provision will be made for some proper supervision, so that much of the perfectly wholesome meat from animals slightly affected with tuberculosis may be utilized and the enormous waste that now results from the total destruction of much of this food may be avoided.

3. It is stated that the Legislature should at once deal with the matter of indiscriminate tuberculin-tests.

In order that this last subject may be made perfectly clear it should be stated that the Cattle Commission under its recent regulations was obliged to take over and pay for all tuberculous animals that were reliably condemned and reported to them, whether the examination was made by their own agents or by others. A considerable number of cattle-owners in Massachusetts have thought that it was to their advantage to employ private veterinarians to test their cattle and report the tuberculous ones to the Cattle Commission for condemnation and appraisement. They seem to do this in preference to having all the work done by the Cattle Commission, to avoid delay. The result is that during the present year $160,000 of the $250,000 appropriated for this purpose had been expended up to the date of the report, and if the Commission were compelled to kill all reacting animals and allow compensation for them, the remainder of the appropriation would soon be exhausted, and as the Commission cannot exceed its appropriation, all of their work must stop when the money gives out. In order to enable the Cattle Commission to more fully govern the expenditure of the appropriation made to it the committee recommends the passage of an act that provides that all testing of cattle with tuberculin when compensation is expected shall be limited to the Cattle Commission or their authorized agents. This act has already become a law, although the minority of the committee opposed it vigorously.

**Louisiana, Georgia, Alabama, Mississippi, North Carolina, Texas, South Carolina and Kentucky will be represented.**

This report has been referred to in the general press, and many unfounded deductions as to the accuracy and reliability of tuberculin have been drawn from it. A careful analysis of the report shows that of the 130 animals condemned as tuberculous and killed, 128 were condemned by tuberculin, one was condemned upon physical examination, and in reference to the other there seems to be a difference of opinion; but as a matter of fact the animal was not condemned at all, but was sent to the abattoir with the other cattle by mistake, and therefore should be disregarded in the compilation of the result. The cow condemned on physical examination was found to have a number of fistulous tracts passing from the stomach through the diaphragm into the thoracic cavity, and her stomach contained metallic foreign bodies. She was afflicted with a cough, was emaciated, and her symptoms in general were quite similar to those observed in advanced cases of tuberculosis. As this animal was not condemned by the use of tuberculin, it can also be disregarded in the compilation of the results. This leaves 128 cows condemned, of which 126 were shown to be tuberculous, while lesions of tuberculosis were not found in the other two, but the microscopical and bacteriological examination of their tissue has not yet been reported upon.

**What are the Eastern States going to present and do for our Nashville meeting?**

Granting that this examination will prove negative, the errors amount to 1.56 per cent. In reference to the other series, it is not possible to form an opinion as to the accuracy of tuberculin, on account of the interval that elapsed between the application of the test and the slaughter of the animals. If, as is reported by some of the experts and by the minority of the committee, four of these animals were probably infected after the test, and, as stated by Dr. Kinnell, one (No. 15) was evidently a recovered case, there remains but one that can be counted as an error of the tuberculin-test, and this means 5 per cent. of error; but in view of the wrong method followed in this portion of the examination, it is not advisable to base a statement as to the accuracy of tuberculin upon it. On the whole, the work justifies everything that has ever been clinically claimed as to the accuracy of tuberculin as a diagnostic agent. It has never been claimed that it is infallible, but its remarkable accuracy has frequently been emphasized, and the results published by the Legislative committee in Massachusetts show it to be more accurate as a diagnostic agent than do the Government reports that have appeared in Germany.

The investigation has developed no new scientific truths, but it has led to the passage of the act providing that all inspections shall be made under the direct supervision of the Cattle Commission, and perhaps it has served other local and private needs that are worth $6000 to the Commonwealth of Massachusetts.

LEONARD PEARSON.

BIOLOGICAL PRODUCTS INDICATED IN VETERINARY PRACTICE.

VETERINARIANS unfamiliar with the recent advances in the production of antitoxins, and who have not acquainted themselves with their methods of use and the results to be sought for, will find in the little monograph issued for veterinarians specially by H. K. Mulford Co., of Philadelphia, one of the most concise, complete, and ready-reference publications that has yet been issued. A request to the company, at their home office, or their branch house in Chicago, will bring this useful little up-to-date book to every reader of the JOURNAL.

